# Hemagglutinin-Specific Non-neutralizing Antibody Is Essential for Protection Provided by Inactivated and Viral-Vectored H7N9 Avian Influenza Vaccines in Chickens

**DOI:** 10.3389/fvets.2019.00482

**Published:** 2020-01-09

**Authors:** Zenglei Hu, Jiangyan Zhao, Yiheng Zhao, Xuelian Fan, Jiao Hu, Lei Shi, Xiaoquan Wang, Xiaowen Liu, Shunlin Hu, Min Gu, Yongzhong Cao, Xiufan Liu

**Affiliations:** ^1^Institutes of Agricultural Science and Technology Development, Yangzhou University, Yangzhou, China; ^2^Joint International Research Laboratory of Agriculture and Agri-Product Safety, The Ministry of Education of China, Yangzhou University, Yangzhou, China; ^3^Animal Infectious Disease Laboratory, School of Veterinary Medicine, Yangzhou University, Yangzhou, China; ^4^Jiangsu Co-innovation Center for Prevention and Control of Important Animal Infectious Diseases and Zoonoses, Yangzhou University, Yangzhou, China

**Keywords:** H7N9 avian influenza, inactivated vaccine, viral-vectored vaccine, non-neutralizing antibody, protection

## Abstract

Hemagglutination inhibition (HI) and virus neutralization antibody (nAb) do not always correlate with the protection of H7 avian influenza vaccines in mammals and humans. The contribution of different classes of antibodies induced by H7N9 vaccines to protection is poorly characterized in chickens. In this study, antibody responses induced by both inactivated and viral-vectored H7N9 vaccines in chickens were dissected. Chickens immunized with inactivated H7N9 vaccine showed 50% seroconversion rate and low HI and nAb titers at week 3 post immunization. However, inactivated H7N9 vaccine elicited 100% seroconversion rate in terms of high levels of HA-binding IgG antibody determined by ELISA. Despite inducing low levels of nAb, inactivated H7N9 vaccine conferred full protection against H7N9 challenge in chickens and markedly inhibited virus shedding. Similarly, Newcastle disease virus (NDV)-vectored H7N9 vaccine induced marginal HI and nAb titers but high level of IgG antibody against H7N9 virus. In addition, NDV-H7N9 vaccine also provided complete protection against H7N9 challenge. Chicken antisera had a high IgG/VN ratio, indicating that a larger proportion of serum antibodies were non-neutralizing antibody (non-nAb). More importantly, passive transfer challenge experiment showed that non-neutralizing antisera provided partial protection (37.5%) of chickens against H7N9 challenge, without significant difference from that provided by neutralizing antisera. In conclusion, our results suggest that antibodies measured by the traditional HI and virus neutralization assays do not correlate with the protection of inactivated and viral-vectored H7N9 vaccines in chickens, and HA-binding non-nAb also contributes to the protection against H7N9 infection. Total binding antibody can be used as a key correlate to the protection of H7N9 vaccine.

## Introduction

Avian influenza A (H7N9) virus emerged as a public health concern in 2013 in China (1). Recently, the fifth wave of H7N9 epidemic hit China, causing significantly more cases of human infection than in the previous four waves (2). During the fifth wave, highly pathogenic (HP) avian influenza H7N9 viruses harboring a polybasic cleavage site (with a “KRTA” insertion) in the hemagglutinin (HA) have emerged, which poses a severe challenge for both poultry industry and public health (3, 4). Unexpectedly, human cases of H7N9 infection and the isolation rate of H7N9 viruses in poultry dropped sharply since October 2017, due to the implementation of the national H7 influenza vaccination program in poultry in September 2017 (5). However, HP H7N9 viruses become well adapted and lethal to ducks, suggesting that H7N9 viruses are not eradicated in poultry and adaption of H7N9 viruses in waterfowls may forecast the next pandemic (5).

In response to the threat of H7N9 influenza, different types of vaccine candidates have been generated, including whole-virus inactivated vaccines, subunit vaccines, virus-vectored vaccines and live attenuated vaccines (6, 7). However, some studies have consistently shown that antibody immunity induced by H7N9 vaccines is qualitatively different from that observed for other influenza subtypes (H1N1, H3N2, and H5N1), which is characterized by low hemagglutination inhibition (HI) or virus neutralization antibody (nAb) but high levels of specific IgG antibody (8–11). This indicates that a large proportion of serum antibodies are non-neutralizing (non-nAb) antibodies. Several studies in mice have demonstrated that non-nAb induced by H7N9 vaccines as well as non-neutralizing H7N9 mAbs are protective *in vivo* (10–13). This indicates that the virus neutralization activity of antibody does not necessarily correlate with protection and other branches of antibody immunity may also contribute to protection.

nAb can inhibit virus infection through various mechanisms, such as blocking virus entry by targeting the receptor binding site, blocking membrane fusion mediated by the HA stem, and inhibiting virus budding from the infected cells. Therefore, nAb is accepted as a primary correlate to the protection of influenza vaccines. However, non-nAb, whose functions have usually been neglected by researchers, is also potent in protection. Fc-dependent immune effector functions associated with non-nAb, including antibody-dependent cellular cytotoxicity (ADCC), antibody-dependent cellular phagocytosis (ADCP) and antibody-dependent complement-mediated lysis (ADCML), are robust in clearing virus particles and virus-infected cells (11, 12).

Inactivated vaccines are widely used in poultry for prevention and control of avian influenza. Antibody titers determined by the traditional serological assays, such as HI and virus neutralization (VN) assays, are generally used as a surrogate for vaccine efficacy. However, the contribution of other classes of antibodies induced by H7N9 vaccines to protection in chickens remains obscure.

In this study, antibody immunity induced by inactivated and Newcastle disease virus (NDV)-vectored H7N9 vaccines in chickens was dissected. Antibody response, including HI antibody, nAb and HA-specific IgG antibody, and vaccine efficacy against challenge with HP H7N9 virus were analyzed. To evaluate the contribution of HA-specific non-neutralizing antibodies to protection, a serum transfer model in chickens was developed.

## Materials and Methods

### Ethics Statement

All animal experiments were approved by the Jiangsu Administrative Committee for Laboratory Animals (Permission number: SYXK-SU-2007-0005), and complied with the guidelines of Jiangsu laboratory animal welfare and ethics of Jiangsu Administrative Committee of Laboratory Animals. Experiments involving live H7N9 strains were performed in animal biosafety level-3 facilities.

### Viruses, Cells, Plasmids, and the HA Proteins

The recombinant NDV-H7N9 vaccine (AI4-GDHA) expressing the H7 HA gene as well as the vector virus (AI4) were generated previously (14, 15). HP H7N9 virus (A/chicken/Guangdong/GD15/2016, GD15) strain was described elsewhere (16). The isolate ID of this strain in Global Initiative on Sharing All Influenza Data (GISAID) is EPI_ISL_305597. Viruses were propagated in 9–11 days old specific-pathogen-free (SPF) embryonated chicken eggs (ECEs). 293T and Madin-Darby canine kidney (MDCK) cells were cultured in Dulbecco's Modified Eagle Medium (DMEM) supplemented with 10% fetal calf serum (FCS) at 37°C, 5% CO_2_. Chicken embryo fibroblasts (CEF) were grown in M199 medium supplemented with 4% FCS. Since H9N2 avian influenza viruses donate the whole internal gene cassette to most H7N9 isolates in the field (17), six plasmids based on pHW2000 vector encoding the six internal genes of H9N2 virus generated previously (18) were used for H7N9 virus rescue. The HA protein of GD15 was expressed in baculovirus expression system in our lab and used as the homologous antigen to measure IgG titers. The HA and HA1 (mainly the globular head region) proteins of H7N9 (A/Anhui/2/2013, AH13) were purchased from Sino Biological (Sino Biological, Beijing, China).

### Generation of the Reassortant H7N9 Virus

The open reading frames of the HA and neuraminidase (NA) genes of GD15 strain were amplified and ligated into pHW2000 vector as described previously (19). To attenuate HP H7N9 GD15 strain, the four amino acids “KRTA” in the cleavage site of HA was deleted using QuikChange Lightning Site-Direct Mutagenesis Kit (Agilent, La Jolla, CA, USA). The reassortant virus was generated by reverse genetics as described elsewhere (20). In brief, equal amount of 293T and MDCK cells (2.5 × 10^5^ each) were mixed and seeded in 6-well plate. Two plasmids encoding the HA and NA genes of H7N9 virus and the six plasmids encoding the internal genes of H9N2 virus were co-transfected into the cells (400 ng each) using the Polyfect transfection reagent (QIAGEN, Hilden, Germany). At 6–8 h after transfection, N-p-Tosyl-L-phenylalanine chloromethyl ketone (TPCK)-treated trypsin was added into the transfection culture at the concentration of 1 μg/mL. At 48 h after transfection, the supernatant and cells were harvested and 0.5 mL of the mixture was inoculated into the allantoic cavity of 9-day-old SPF ECEs. The presence of virus was confirmed using hemagglutination (HA) test. The reassortant virus was designated as rGD15. Deletion of “KRTA” in the virus was confirmed by sequencing. Virus titer and virulence of H7N9 viruses were assessed in ECEs.

### Preparation of Inactivated H7N9 Vaccine

rGD15 was propagated in 10-day-old SPF ECEs and at day 4 post inoculation, the allantoic fluids were harvested and 10 mL of the allantoic fluids were inactivated by treatment with formaldehyde at the ratio of 1:1,000 to the allantoic fluids according to the procedure recommended by World Organization of Animal Health (OIE) (https://www.oie.int/fileadmin/Home/eng/Health_standards/tahm/3.03.04_AI.pdf). The inactivation mixture was incubated on a rotating incubator at 4°C for 48 h. The treated allantoic fluid was inoculated into ECEs for three rounds and virus inactivation was confirmed by HA test. The inactivated antigen was then mixed with mineral oil adjuvant (Seppic MONTANIDE^TM^ ISA 71R VG) at the ratio of 1:2 for emulsion to make a water-in-oil vaccine formulation. The inclusion level for each chicken was 512 HA unit (HAU). Meanwhile, the allantoic fluids collected from non-inoculated SPF ECEs were also emulsified for use as the sham control.

### Immunization and Challenge Study of H7N9 Vaccines in Chickens

Experiment I: Ten 4-week-old SPF white leghorn chickens were intramuscularly immunized with 0.3 mL of rGD15 vaccine (512 HAU). Five chickens were immunized with the oil-emulsified product containing the allantoic fluids from non-inoculated eggs as the sham control. At weeks 2 and 3 post immunization (pi), chickens were bled to collect serum samples for serological tests. At week 3 pi, birds were intranasally inoculated with 10^5^ 50% embryo infectious dose (EID_50_) of HP H7N9 GD15 strain. Clinical signs of chickens were monitored daily and clinical scores were given for each chicken based on the following range: 0-no clinical signs; 1-mild clinical signs, such as reluctance to move, respiratory signs, and diarrhea; 2-sever clinical signs, such as nervous disorders; 3-dead. At days 5 and 7 post challenge (pc), laryngotracheal and cloacal swabs were collected to monitor virus shedding according to a published protocol (21).

Experiment II: Twenty 2-week-old SPF chickens were randomly divided into three groups. Birds in group I (*n* = 10) were immunized via intranasal and eye-drop routes with 10^6.0^ EID_50_ of AI4-GDHA. Chickens in group II (*n* = 5) were inoculated with 10^6.0^ EID_50_ of the vector virus AI4 through the same route. Chickens in group III (*n* = 5) were inoculated with 100 μL of PBS as the sham control. At weeks 2, 3, and 4 pi, chickens were bled and serum samples were taken for determination of antibody titers. At week 4 pi, all chickens were challenged with 10^5.0^ EID_50_ of HP H7N9 GD15 strain via intranasal and eye-drop routes. Clinical signs were monitored on daily basis for 14 days and scores were given to each bird according to the standards described above.

### Serological Tests

HI antibody titers of antisera obtained from chickens immunized with inactivated and vectored H7N9 vaccines were measured according to the standard OIE procedure. In addition, nAb titers of antisera were determined as described previously and HP H7N9 GD15 strain was used as the antigen (11). HA-specific IgG titers were measured as described previously (9) with some modifications. Herein, ELISA plates were coated with 0.35 μg/mL of the purified HA protein of GD15 overnight at 4°C. The plates were washed three times with PBS containing 0.5% Tween 20 (PBST) and then blocked with 5% skim milk in PBST for 1 h at room temperature (RT). The plates were washed three times with PBST and then incubated with serial dilutions of the serum samples at 37°C for 1 h. After washing, the plates were incubated for 1 h with horseradish peroxidase (HRP)-conjugated goat anti-chicken IgY antibody at 37°C (Southernbiotech, Birmingham, AL, USA). The plates were washed three times with PBST and developed with 3,3,5,5'-Tetramethylbenzidine (TMB) as the substrate (Beyotime, Nantong, China). After 10 min of incubation at RT, the plates were read at 370 nm. The endpoint titer of the sera was defined as the highest dilution at which the mean OD value of duplicate wells were >2-fold above the mean OD value plus 2 standard deviations for the sera from the sham control chickens. In addition, to identify the HA region recognized by chicken immune sera, the HA and HA1 proteins of H7N9 AH13 strain were used as the coating antigens in ELISA. The experiment was performed as described above.

### Passive Transfer Challenge Experiments in Chickens

To evaluate the contribution of non-neutralizing serum antibody to protection, a serum transfer and challenge study in chickens was developed. Sera obtained from chickens immunized with whole-virus inactivated H7N9 vaccine contain antibodies against the NA protein, which also contributes to protection against avian influenza. Therefore, to investigate the role of HA-specific antibody, chicken antisera obtained from animals immunized with NDV-H7N9 vaccine were used in this experiment. Briefly, pre- and post-immunization (4 week) serum samples from NDV-H7N9-vaccinated birds were pooled. In addition, serum samples with high HI/VN titers (HI/VN^+^) collected from chickens immunized with inactivated H7N9 vaccine were used as the positive control. Eight 7-day-old chickens were administrated intravenously with the pooled serum (50 μL per bird) and five chickens were inoculated with pre-immunization or the positive serum samples via the same route. Another five chickens were inoculated with PBS as the sham control. After 2 h, birds were intranasally inoculated with 10^5^ EID_50_ of H7N9 GD15 strain. Morbidity and mortality were monitored daily for a period of 14 days.

### Statistical Analysis

Statistical analysis of the data in our study was performed using Graphpad prism 8.0 software. Correlation analysis between HI&VN, HI&IgG, and VN&IgG was performed using Pearson regression method. Survival of the animals in the serum transfer study was compared using Mantel-Cox test. *p* < 0.05 was considered as a significant difference.

## Results

### Generation of the Reassortant H7N9 Virus

The GD15-derived reassortant virus (rGD15) with the deletion of “KRTA” from the cleavage site was generated. The mean death time (MDT) for embryos of the wild-type GD15 strain was 36 h, indicating that it is a typical highly pathogenic virus (Table 1). The reassortant virus rGD15 was not lethal to chicken embryos within 5-day incubation (MDT > 120 h) (Table 1), suggesting that GD15 was remarkably attenuated. The reassortant virus had comparable virus titers as the wild type virus in ECEs. Sequencing of the viruses at different passages confirmed the “KRTA” deletion in the HA gene (data not shown). These data suggested that HP H7N9 virus was significantly attenuated by modifying the HA cleavage site and the attenuated virus can be used as the master seed virus for inactivated vaccine.

**Table 1:** Biological characterization of the reassortant H7N9 virus.

**Virus**	**Virulence**	**Virus titration**	
	**MDT^**a**^(h)**	**HA titer^**b**^ (log_**2**_)**	**EID_**50**_/mL^**c**^ (log_**10**_)**
GD15	36	8	9.63
rGD15	>120	9	9.50

a *Mean death time of chicken embryos*.

b *HA, hemagglutination*.

c *EID_50_, 50% embryo infectious dose*.

### Dissection of Antibody Response Elicited by Inactivated H7N9 Vaccine in Chickens

**Figure 1:**
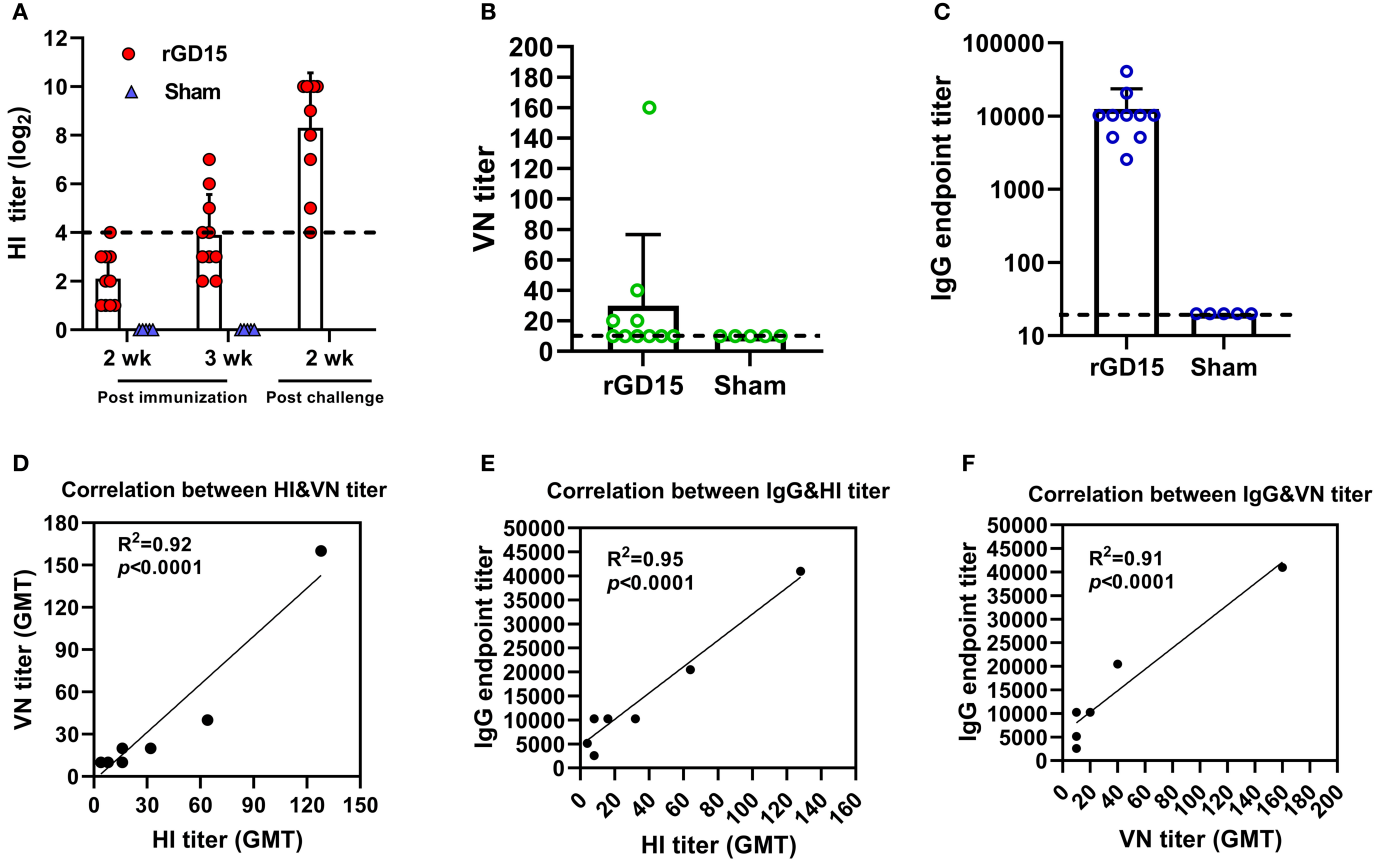
Antibody response induced by inactivated H7N9 vaccine in chickens. **(A)** HI, **(B)** VN, and **(C)** IgG titers induced by the vaccine. **(D)** Correlation analysis between VN and HI antibody titers. **(E)** Correlation analysis between IgG and HI antibody titers. **(F)** Correlation analysis between IgG and VN antibody titers. The dotted line in **(A)** indicates the cutoff value for positive reading in HI assay (4 log_2_) and the lines in **(B,C)** stand for the lower detection limit for serological assays. GMT in **(D–F)** stands for geometry mean titer.

For the inactivated H7N9 vaccine immunization study, at week 2 pi, only one rGD15-immunized chicken seroconverted based on the cutoff value for HI assay (HI ≥ 4 log_2_) and the mean HI titer was 2 log_2_. At week 3 pi, seroconversion rate increased to 50% and the mean HI titer was 4 log_2_. HI titers increased sharply in survivors after H7N9 challenge, indicating the presence of anamnestic responses (Figure 1A). No HI antibody response was detected in the sham control birds. Similarly, the rGD15 vaccine also induced low seroconversion rate (40%) based on VN titers and the mean VN titer was around 30 (Figure 1B). However, in fact, the vaccine induced 100% seroconversion rate in terms of a robust IgG antibody response in each chicken measured by ELISA, and the mean IgG titer was 10,000 (Figure 1C).

To gain insights into the relationship between HI, VN, and IgG titers, correlation analysis was performed. There were good correlations between HI&VN, HI&IgG, and VN&IgG titers for the rGD15 vaccine (Figures 1D–F), indicating that functional antibodies were proportional to the total binding antibodies for inactivated H7N9 vaccine. More importantly, we estimated the proportion of non-nAb by using a previously defined surrogate measure, i.e., the ratio of ELISA(IgG) to VN titers (22). We found that the percentage of nAb in total HA-binding antibodies was only 0.2% and the overwhelming majority of H7 HA-specific antibodies in serum were non-nAbs (**Figure 3A**).

Taken together, these results suggested that although inactivated H7N9 vaccine induced low HI and VN antibodies in chickens, it is actually highly immunogenic in terms of elicitation of high levels of IgG titers. Additionally, non-nAb was dominant in serum antibodies after immunization with inactivated H7N9 vaccine.

### Dissection of Antibody Response Elicited by NDV-H7N9 Vaccine in Chickens

**Figure 2:**
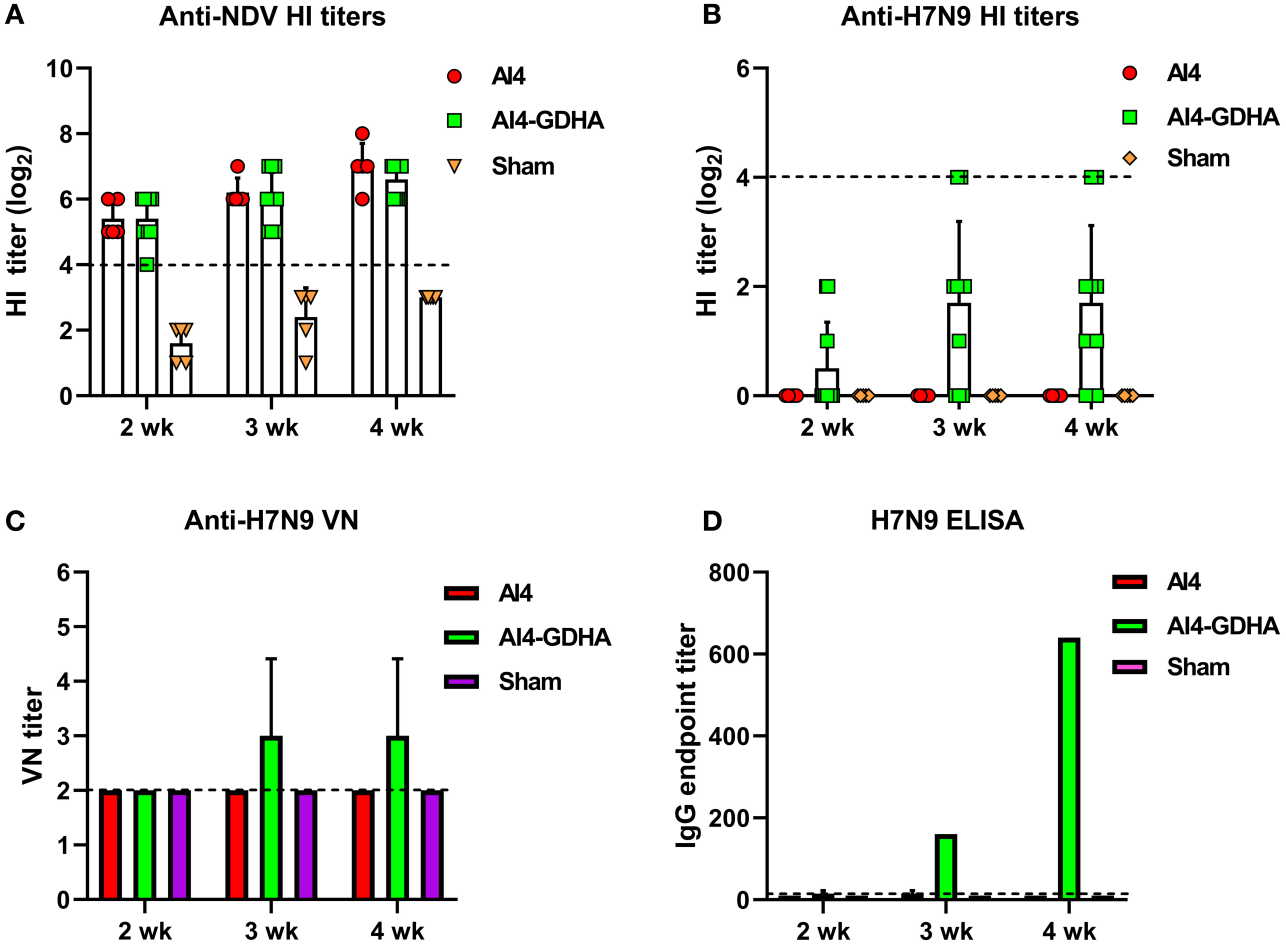
Antibody response induced by NDV-vectored H7N9 vaccine in chickens. **(A)** HI titers against NDV vector. **(B)** HI titers against H7N9 virus. The dotted lines in **(A,B)** indicate the cutoff value for positive reading in HI assay (4 log_2_). **(C)** VN antibody titers against H7N9 virus. **(D)** IgG titers determined by ELISA. Serum samples from individual chicken were pooled for measurement of VN and IgG titers. The dotted lines in **(C,D)** stand for the lower detection limit for VN test and ELISA.

**Figure 3:**
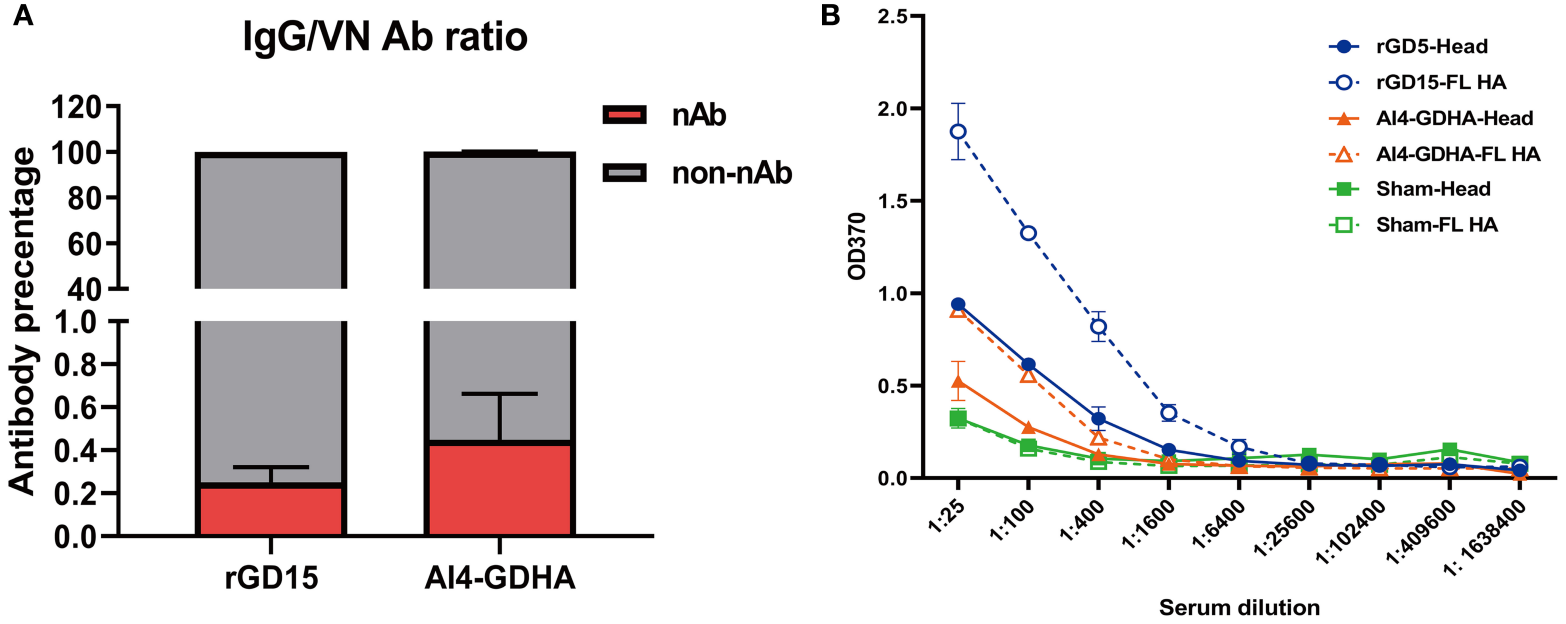
Ratio of nAb and non-nAb in immune sera and the HA region recognized by the sera. **(A)** Ratio of nAb and non-nAb in sera was calculated. **(B)** IgG titers against the HA and HA1 proteins. FL, the full-length HA protein; Head, the HA1 protein; NC, the sham control serum. Serum samples from each immunized chicken were pooled and tested.

Antibody response induced by viral-vectored vaccine was also analyzed. NDV-H7N9 vaccine elicited a robust HI antibody response to NDV vector (Figure 2A), whereas only a small fraction of the vaccinated chickens (1 or 2 out of 10) developed HI titers against H7N9 and the mean HI titers were very low at different time points (Figure 2B). In addition, the AI4-GDHA vaccine also induced low anti-H7N9 nAb titers in chickens (Figure 2C). In contrast, AI4-GDHA induced high levels of IgG antibody at week 3 and 4 pi (Figure 2D). In addition, a high IgG/VN ratio was also observed in serum of chickens vaccinated with the NDV-H7N9 vaccine (Figure 3A). These findings indicated that NDV-H7N9 vaccine displayed a similar antibody profile as inactivated vaccine in chickens, which was characterized by low HI/VN and high IgG titers.

### Identification of the HA Region Recognized by Immune Serum

Since high levels of non-nAbs were detected in the immunized chickens, this indicates that other regions in HA outside the immunodominant HA head might be targeted by non-nAbs. To address this question, IgG titers against the full-length HA and HA1 proteins were determined by ELISA. The results showed that rGD15 immune serum had higher IgG titers against the HA protein compared to that against the HA1 protein (Figure 3B). Similarly, we also detected higher levels of IgG titers to HA compared to that against HA1 for the sera obtained from chickens immunized with NDV-H7N9 vaccine (Figure 3B). These data suggest that both inactivated and NDV-H7N9 vaccines elicited antibody against both the HA head and stalk regions.

### Efficacy of H7N9 Vaccines in Chickens

**Figure 4:**
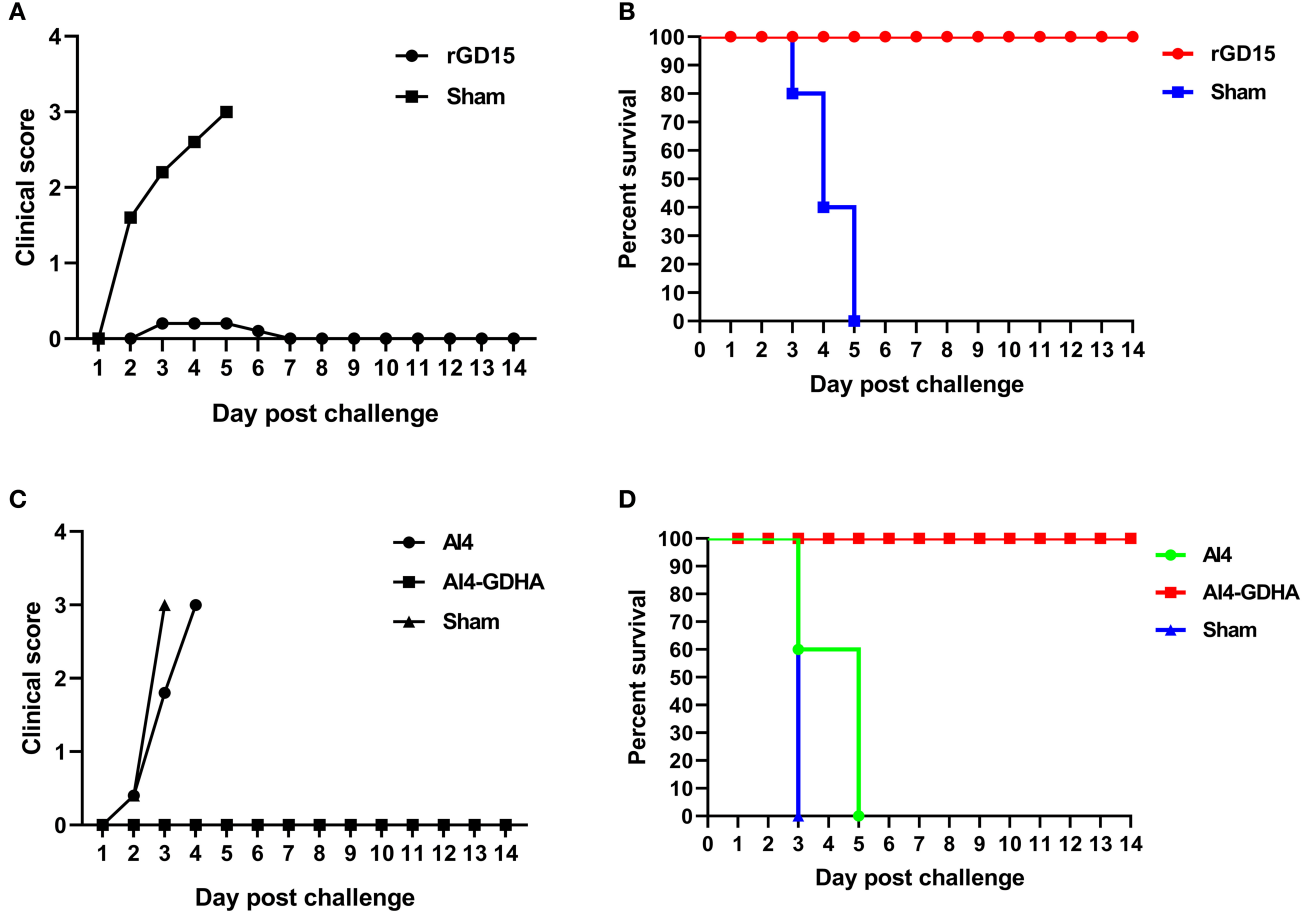
Clinical scoring and survival of animals after H7N9 challenge. **(A)** Clinical scoring and **(B)** survival of chickens immunized with the rGD15 vaccine. **(C)** Clinical scoring and **(D)** survival of chickens immunized with the NDV-H7N9 vaccine.

Chickens immunized with inactivated H7N9 vaccine were challenged with HP H7N9 virus at week 3 pi. All sham-immunized birds displayed severe disease signs and died within 5 days pc, indicating the challenge dose of H7N9 is highly fatal to chickens (Figures 4A,B). Although HI titers in 70% of rGD15-immunized chickens were below 5 log_2_ that is generally considered as the minimum serological titer for protection from mortality, all the immunized chickens survived H7N9 virus challenge (Figure 4B). Only two birds with HI titers of 2 log_2_ exhibited transient mild clinical signs, such as diarrhea and respiratory signs, within 5 days pc (Figure 4A). Moreover, virus shedding was detected in both laryngotracheal and cloacal swabs in two and four rGD15-vaccinated chickens at days 5 and 7 pc, respectively (Table 2). It is noted that chickens shedding virus were not all those with low HI titers, and two birds with HI titers ≥ 4 log_2_ also shed virus. These findings suggested that the rGD15 vaccine conferred a complete protection against H7N9 infection, despite that it induced low HI and nAb titers in some chickens.

**Table 2:** Virus shedding of chickens immunized with inactivated H7N9 vaccine.

	**Proportion of chickens shedding virus**			
	**Day 5 pc**^**a**^		**Day 7 pc**	
**Group**	**Laryngotracheal swabs**	**Cloacal swabs**	**Laryngotracheal swabs**	**Cloacal swabs**
rGD15	2/10	2/10	4/10	4/10
Sham	NA^b^	NA	NA	NA

a *pc, post challenge*.

b *NA, not available due to death of animals*.

In the challenge study for the vector vaccine, we found that all the sham control chickens and birds immunized with the vector AI4 exhibited severe clinical signs and succumbed to death within 5 days (Figure 4C). All chickens immunized with AI4-GDHA survived the challenge and no chickens exhibited any disease signs in the observation period (Figures 4C,D). Similar to the findings for inactivated H7N9 vaccine, although NDV-H7N9 vaccine induced negligible HI or VN antibodies in chickens, the vaccine still provided 100% protection from H7N9 challenge.

### Passive Transfer Challenge Study

**Figure 5:**
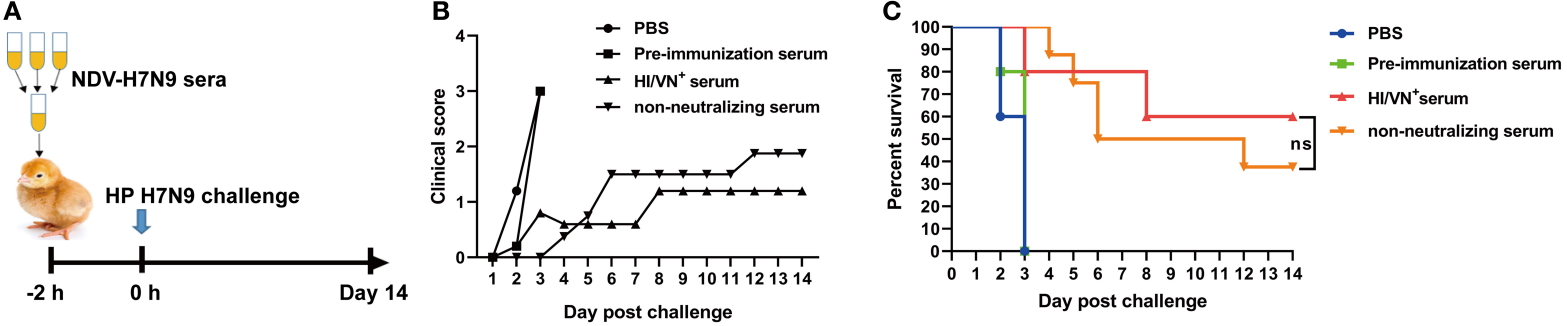
Passive transfer challenge study of non-neutralizing sera. **(A)** Schematic illustration for the serum transfer experiment. **(B)** Clinical scoring and **(C)** survival of chickens after H7N9 virus infection. “ns” in **(C)** indicates non-significant difference.

The protective efficacy of HA-specific non-neutralizing antibodies was determined in a chicken passive transfer challenge model (Figure 5A). We found that all the sham control chickens as well as the animals received pre-immunization sera succumbed to H7N9 infection within 5 days (Figures 5B,C). Three out of five chickens received HI/VN^+^ sera survived H7N9 infection. Moreover, 37.5% survival rate was observed for chickens received non-neutralizing sera obtained from NDV-H7N9-vaccinated animals. Survived chickens in these two groups displayed no clinical signs. Of note, statistical analysis revealed that there was no significant difference in survival rate between HI/VN^+^ sera and non-neutralizing sera (Figure 5C). Passive transfer challenge experiment indicated that H7 HA-specific non-nAb can confer partial protection against H7N9 infection, which was equivalent to HI/VN^+^ antibodies.

## Discussion

Serum antibodies measured by the traditional HI and VN assays are widely accepted as immune surrogates for protection of influenza vaccines. However, the emergence of H7N9 avian influenza provides a timely opportunity to redefine correlates of protection for pandemic influenza vaccines. In this study, we found that both inactivated and viral-vectored H7N9 vaccines exhibited a similar antibody response profile characterized by low HI/VN titers but high levels of HA-binding non-nAb in chickens. This fraction of antibody conferred protection *in vivo* against H7N9 challenge, suggesting that non-nAb is a major contributor to protection provided by both inactivated and vectored H7N9 vaccines. Our findings indicate that HI and VN antibodies are not good correlates for efficacy of H7N9 vaccines and total HA-binding IgG antibody should be used as an important surrogate marker.

HI or VN activity is generally considered as an immune correlate for efficacy of influenza vaccines. For the seasonal influenza vaccines, an HI titer of 1: 40 is recognized as an immunogenic correlate corresponding to a 50% reduction in the risk of influenza virus infection in adults (23). However, for pandemic influenza, such as H7 and H5 subtypes, immune surrogate for vaccine protection has not been established. Our data showed that although inactivated and NDV-H7N9 vaccines induced low HI and VN titers, they still provided complete protection against H7N9 infection (Figures 1, 2, 4). This finding further supports that at least for H7 avian influenza vaccine, HI, and VN antibodies are not good indicators for protection. Our findings support the remarks from some influenza experts (24), highlighting the need to develop new immune correlates for H7N9 vaccines.

On the other hand, in addition to virus neutralization antibody, non-nAb can also provide protection, which is usually overlooked by researchers. In the present study, we demonstrated that a large proportion of serum antibodies elicited by H7N9 vaccines are antibodies without detectable virus neutralization activity (Figure 3), indicating that non-nAb is dominant in antibody immunity against H7N9 vaccines in chickens. In addition, HI nonseroconverters immunized with inactivated H7N9 vaccine as well as all birds vaccinated with NDV-H7N9 vaccine survived H7N9 challenge, suggesting the contribution of non-nAb to protection. Moreover, passive transfer of non-neutralizing sera obtained from NDV-H7N9-immunized animals provided partial protection against virus infection, comparable with sera with high HI/VN activity, which further confirmed the role of non-nAb in protection (Figure 5). Consistently, Kamal et al. have shown that inactivated H7 subtype (H7N9, H7N2, and H7N7) influenza vaccines induced significantly lower HI and VN titers compared to the H1 and H3 subtypes, while they elicited high levels of non-neutralizing IgG antibody and good protection against virus infection (10). Moreover, Ng et al. have revealed that binding antibodies measured by ELISA act as a powerful correlate of protection and to determine seroconversion in a natural pandemic H1N1 virus transmission setting (25). Other studies on therapeutic H7N9 mAb also showed that non-neutralizing mAbs are equivalently efficacious as neutralizing mAbs against H7N9 virus infection (12, 13).

However, although we determined the importance of non-nAb to protection for H7N9 vaccines, there are two main questions to be answered. After H7N9 vaccination, non-nAb is dominant in serum antibodies, which is different from antibody response profile observed for other subtypes. This indicates that the antigenic epitopes in HA recognized by non-nAb may be immunodominant, however, these epitopes have not been identified yet. Herein, comparison between binding with HA and HA1 demonstrated that inactivated and NDV-vectored H7N9 vaccines elicited antibodies against both the HA head and stalk regions (Figure 3B). However, the immunodominant epitopes in H7 HA for non-nAb induction are still unidentified. Additionally, the mechanisms for protection of non-nAb still remain unclear. Some studies have reported that non-neutralizing mAbs or human serum provide protection through Fc immune effector functions, such as ADCC, ADCP, and ADCML (11–13). However, the role of Fc immune effector functions has been poorly profiled in the context of immune serum, both in humans and animal models. Therefore, further studies are required to elucidate the molecular basis and the protection mechanisms for non-nAb in the process of H7N9 immunization.

In summary, our results suggest that apparently low immunogenicity observed for H7N9 vaccines may be at least partly related to measuring antibody titers using the traditional HI and VN tests, which may not provide a true measure of protective immunity associated with H7N9 immunization. HA-specific non-neutralizing antibodies are essential for protection of H7N9 vaccines in chickens. This study underscores the need for inclusion of HA-binding IgG antibody for evaluation of H7 influenza vaccines.

## Data Availability Statement

The datasets generated for this study are available on request to the corresponding author.

## Author Contributions

ZH and XiuL designed the study. ZH, JZ, YZ, and XF conducted the experiments. LS generated the plasmids for generation of the reassortant virus. ZH and JH analyzed the data and drafted the manuscript. XW, XiaoL, and SH reviewed the procedures of the study. MG and YC revised the manuscript. All authors have read and approved the submission of the manuscript.

### Conflict of Interest

The authors declare that the research was conducted in the absence of any commercial or financial relationships that could be construed as a potential conflict of interest.
